# Social Contact and Wellness Outcomes for Older Adults in Different Residential Settings

**DOI:** 10.1093/geroni/igaa057.1310

**Published:** 2020-12-16

**Authors:** Joseph Bihary, Jennifer Smith, Dugan O’Connor, Ajla Basic, Janis Sayer, Cate O’Brien

**Affiliations:** Mather Institute, Evanston, Illinois, United States

## Abstract

More frequent social contact with others has been shown to be associated with positive well-being outcomes for older adults, who may be at increased risk of loneliness or isolation. The current study investigates whether the potential benefits of social contact might vary by social contact medium and by residence type (senior living (SL) settings vs. the broader community). 5,148 older adults residing in SL communities completed surveys on health and well-being. Data were combined with responses from 1,000 demographically similar older adults residing outside SL settings who participated in a similar study. Participants in both studies reported their frequency of social contact with friends (4 types: in-person meet-ups, speaking by phone, writing or emailing, and communicating via social media), subjective overall health, and life satisfaction. Results of multilevel regression analyses revealed that more frequent in-person social contact with friends was associated with more positive ratings of overall health only for participants in non-SL settings, but not those in SL. More frequent in-person social contact was also associated with higher life satisfaction, and this effect was stronger for non-SL participants. More frequent contact by phone and written letters/emails was associated with better ratings of overall health, regardless of residence type. In contrast, more frequent social media use was associated with lower ratings of overall health. Analyses controlled for age, gender, education, marital status, income, chronic health conditions, and depressive symptoms. The observed patterns of results speak to the possible protective benefits of social engagement for older adults in different residence settings.

